# Psychological Profile and Competitive Performance in Group Aesthetic Gymnastics

**DOI:** 10.3389/fspor.2021.625944

**Published:** 2021-02-19

**Authors:** Celia Armada Martínez, Francisco Cavas-García, Arturo Díaz-Suárez, Alfonso Martínez-Moreno

**Affiliations:** ^1^Master of Research in Physical Activity and Sport Sciences, Faculty of Sport Sciences, University of Murcia, Murcia, Spain; ^2^Department of Physical Activity and Sport, Faculty of Sport Sciences, University of Murcia, Murcia, Spain

**Keywords:** sport, competitive anxiety, optimism, motivational climate, cohesion

## Abstract

The objective of the research was to evaluate the perceptions about the psychological variables of cohesion and motivational climate that AGG gymnasts have about the context created by the coaches in their teams and to analyze in the different categories the optimism and competitive anxiety they face in competitive situations. In an attempt to clarify the psychosocial training patterns in this sport and the influence they have on the well-being of its practitioners, competitive anxiety, motivational climate, team cohesion and level of optimism were taken as dependent variables. The sample was made up of 98 national and international junior and senior athletes in the aesthetic gymnastics group aged 13 to 27 (M = 17.1; SD = 2.8). The Perceived Motivational Climate in Sport Questionnaire for motivational climate, the Sport Anxiety Scale-2 for competitive anxiety, the Group Environment Questionnaire for cohesion and the Revised Life Orientation Test for optimism were used in the evaluation. The results obtained show a positive and statistically significant correlation between anxiety and ego involvement, while, for task involvement, high levels of cohesion are associated with high levels of optimism (*p* = 0.005). In conclusion, the data confirm that high levels of cohesion are associated with high levels of optimism, in addition high scores on task involvement show high scores on cohesion and optimism, however high scores on ego involvement are associated with low scores on cohesion and optimism.

## Introduction

Sporting competition requires challenges and demands resilience from athletes, to adapt to change and overcome difficult situations. The Aesthetic Group Gymnastics (AGG) assists with the execution of the choreographies performed by groups of 6 to 12–14 gymnasts, depending on the category. The choreographies must include the mandatory elements (waves, swings, jumps, balances, and so forth) that are specified in the scoring regulations (IFAGG, 2017).

Sport generates high levels of stress and anxiety (Hanton et al., 2015). Practicing and employing a variety of psychological strategies to combat possible negative emotional states, such as sports-related anxiety, has become part of the training of athletes (Weinberg and Gould, 2015). Anxiety is a transient emotional state manifested by feelings of subjective tension and apprehension, which cause an increase in the autonomous nervous system activity linked to brain function and anchored to basic psychological processes, such as attention, perception, memory, emotion and thought (Spielberger and Guerrero-Díaz, 2002). Understanding that competitive anxiety is a context-specific distress that would appear systematically before or during the competition, from a perspective of the Theory of self-determination, it should be considered an indicator of contextual distress (Ramis et al., 2017). The theory of drive proposes the relationship between performance and state anxiety as linear, with greater anxiety leading to better performance (Hull, 1943). Athletes tend to perceive stressful situations as challenges and opportunities for growth and development (García-Secades et al., 2014).

Anxiety is also related to sociodemographic variables such as age and gender in sports contexts, as it is multifactorial (González-Hernández et al., 2020). Less experienced athletes tend to have greater uncertainty due to the unknown, which generates higher levels of pre-competition anxiety (Vaca et al., 2017). Athletes with lower levels of anxiety perform better in competition while those with high levels of anxiety perform worse (Ngo et al., 2017). Other authors such as Woodman and Hardy (2003) indicate a significant negative association between anxiety and competitive performance. Rocha and Osório (2018) conclude from their meta-analysis that athletes with less competitive experience have higher levels of anxiety than those with more competitive experience.

In the field of sport, the concept of motivational climate reflects the structure of objectives and situations of the environment generated by important people (coaches, parents) accepting the predictive value for sport-related results (Harwood et al., 2015). Motivation is one of the most studied psychological factors in sports psychology and by sports performance researchers (Castro-Sánchez et al., 2019a,b). Studies agree that the type of climate that coaches create may be related to the perceptions of their athletes and the cohesion of the team (Heuze et al., 2006), although this factor depends to a large extent on the ages of the athletes, where older athletes appear to be more influenced by the model transmitted by the coach (Gardner et al., 1996). Already predicted by Duda (2001) is the climate-cohesion motivation relationship based on the structural components of the climate created by the coaches. This was later contrasted with substantial empirical evidence showing that perceptions of a climate involving tasks are associated with a more adaptive pattern of achievement (Duda and Balaguer, 2007). Heuze et al. (2006) demonstrated the existence of links between the motivational climate, perceived team cohesion and collective effectiveness. When the coach and the athlete work in a positive motivational climate, group cohesion also improves (Heuze et al., 2018).

The cohesion of a sports team is understood as a dynamic process reflected in the group's tendency to remain united in the search for an objective that meets the affective needs of the members (Carron et al., 1985). Cohesion involves both individual and group components, respecting individual differences and/or potentialities and perceptions concerning the team's task and commitment to social unity (Carron et al., 1985; Horn et al., 2012). Cohesion is often identified as a simple relationship of friendship, but in a group, it is important to verify the orientation of the task in order to achieve a determined and inspiring goal shared by all team members (Di Onofrio et al., 2019). The findings of Prapavessis and Carron (1996) revealed that cohesion and anxiety were associated. Furthermore, Eys et al. (2003) indicate that being part of a cohesive group leads to greater group effectiveness. Cohesion has been shown to be a determining factor in collective sports such as football, basketball and handball (Galatti et al., 2015; Olmedilla et al., 2015; Ureña et al., 2015). In a group setting, one variable that is consistently related to perceptions of cohesion is the motivational climate (McLaren et al., 2017).

Some authors such as West et al. (2009) already indicated that it is necessary to identify relevant determinants for specific groups and goals (or outcomes), such as team optimism. The Psychology of Positive Emotions theory proposes that intelligent optimism is the ability to face adversity as a challenge and not a threat, thereby turning crises into opportunities (Avia and Vázquez, 1998). In terms of performance sport, it highlights two significant variables associated with successful athletes, namely optimism and competitiveness (García-Naveira and Díaz, 2010; De la Vega et al., 2012; Ortín et al., 2013). Seligman et al. (1990) investigated the relationship between optimism and athletic performance and found that optimistic people matched and even improved their performances after an unfavorable performance, and these results were strengthened in subsequent studies (Martin-Krumm et al., 2003; Ortín et al., 2011). Optimism is not only a cold cognition, but it is also motivational and motivating (Urrea, 2015). Athletes tend to exhibit greater mental strength than non-athletes, which contributes positively to optimism and sports resilience (Guillén and Laborde, 2014; Reche et al., 2014; Cowden et al., 2016). Metal strength is divided into three aspects: Physical (Physical Self-Concept), Emotional (Motivation and Sporting Commitment), and Mental (Resilience and Optimism) helping the athlete to develop their capabilities as a whole in order to achieve sporting success (Carr, 2010).

Most scientific studies on gymnastics, especially concerning the modalities of rhythmic gymnastics and artistic gymnastics, are based on anthropometric profiles and body composition (Carter and Heatch, 1990; Carter and Ackland, 1994; Drinkwater and Mazza, 1994; Norton and Olds, 1996; Yústiz et al., 2015); type of complexion (Lisitskaya, 1995); technical-tactical and specific performance factors (Lisitskaya, 1995; Fasting et al., 2000; Fernández, 2001; Martínez-Gallego, 2004); energy requirements, limiting factors and fatigue associated with training and competition (Terrados et al., 2011); analyses of the effects of competition on retired rhythmic gymnasts (Mendizábal, 2000); burnout and optimism in rhythmic trainers (Saquero et al., 2018); nutritional, anthropometric, and psychological aspects of rhythmic gymnastics (San Mauro et al., 2016); case studies on psychological interventions in rhythmic gymnastics (Álvarez-Solves et al., 2013) and psychological training in elite sports gymnastics (Fournier et al., 2005).

From the psychosocial point of view of groups in the sporting context, numerous studies have been carried out that emphasize the importance of exploring and explaining the relationships established between group variables and how they affect individual performances and well-being within the team (Duda and Balaguer, 2007; Ortín et al., 2011; García-Naveira et al., 2015; Suárez, 2018). However, within AGG, there are no studies, to the best of the authors' knowledge, that have analyzed all these variables.

This makes us consider the following objectives: (i) to know the psychological variables of cohesion and motivational climate of GGA gymnasts according to categories; (ii) analyze the optimism and competitive anxiety faced by gymnasts, according to marital status, educational level and different categories, in competitive situations; (iii) to determine the effect of the variables, category and years competing, as well as the motivational climate, group cohesion and optimism on competitive anxiety. This allows us to make the following hypotheses (h1) The lower the sports category, the greater the anxiety, (h2) The greater the optimism, the less the anxiety.

## Materials and Methods

### Sample

Two-hundred twenty gymnasts who participated in the First Phase of the Spanish Group Aesthetic Gymnastics Cup (2018–2019) in the junior (13–16 years) and senior (>16 years) categories were considered for inclusion. The final sample consisted of 98 female gymnasts, aged between 13 and 27, with an average age of around 17 years old (M = 17.1; ST = 2.8). In this sample, 44.05% of the gymnasts were in the junior category (13–16 years), with the remainder, or 55.95%, in the senior category (>16 years). The gymnasts had been competing for an average of 8.7 years (SD = 3.4). In addition, 86% of the gymnasts were single, and 14% were married. Just over half (51%) of the gymnasts had received a secondary education, 30.2% had received a primary education and 18.8% other qualifications. The number of hours that the gymnast trained was similar, namely 6–12 h per week, distributed across 4–6 sessions of 2.5 to 3 h in duration.

### Instruments

To measure the motivating climate level, we used the second version of the Perceived Motivational Climate in Sport Questionnaire (PMCSQ-2) prepared by Newton et al. (2000), which was validated in Spanish by Balaguer et al. (1997). It consists of two correlated variants that measure the degree to which athletes perceive that the coach creates a climate of involvement in the task or a climate of involvement in the ego. The PMCSQ-2 questionnaire for set sports consists of 29 items: 15 oriented toward the climate of involvement in the task, and 14 oriented toward ego involvement. The response format consists of a five-point Likert scale ranging from 0 for “strongly disagree” to 4 for “strongly agree.” Internal consistency analyses found 0.80 Alpha Cronbach (α) coefficients for both the climate scale involved in the task and for the climate scale involved in the ego.

To measure the anxiety-specific trait in sports competition, we used the Sport Anxiety Scale-2 (SAS-2) (Smith et al., 2006) adapted for Spanish by Ramis et al. (2010). The questionnaire has 15 items divided into three subscales with 5 items each: Somatic Anxiety (AS), Concern (P), and Deconcentration (D). Each item is answered with a score from a 4-point Likert scale ranging from 1 (nothing) to 4 (much). Total scores per subscale are obtained from the sum of the scores of the items within the subscale and can range from 5 to 20, where, for example, a low anxiety score means that there is a low probability that a person will be anxious, and a high score indicates a tendency to become anxious in a competitive situation. The total score, which measures competitive sensitivity, is the sum of the scores for all items and ranges in value from 15 and 60. In terms of consistency, the α for the somatic scale was 0.83, whereas it was 0.78 for the worry scale and 0.73 for the deconcentration scale.

To assess group cohesion in sports environments, the Spanish adaptation of the Group Environment Questionnaire (GEQ) (Carron et al., 1985) developed by García-Calvo (2006) was used. It consists of 12 positive elements, grouped into four factors with three items each: Social Group Attraction (AGS), Group Attraction to Task (AGT), Social Integration (IS) and Integration to the Task (IT). Total group cohesion is calculated with the sum of the scores of all items. Each item is measured on a 9-point Likert scale, ranging from strongly disagree (1) to totally agree (9). Both the dimensions and the global cohesion factor (GEQ) have α's above 0.70.

The Revised Life Orientation Test (LOT-R) developed by Scheier et al. (1994) was used to measure the level of dispositional optimism or widespread predisposition toward expectations of positive outcomes (Otero et al., 1998), validated by Ferrando et al. (2002) to the spanish language. Composed of 10 items, in which subjects indicate the degree of agreement or disagreement with statements such as “in difficult times, I generally hope for the best,” it uses a scale of 5 points, where 0 corresponds to “strongly disagree,” and 4 corresponds to “strongly agree.” Four of the items are used for control or fill, three items are skewed positive (optimistic) and three are skewed negative (pessimistic). With regard to the correctness and interpretation of the test, there are two options (Ferrando et al., 2002): on the one hand, each skew can be measured separately; on the other hand, total optimism can be measured by reversing the scores of the items drafted with a negative skew. The coefficients of internal consistency were 0.75 for optimism and 0.69 for pessimism. Different works have supported working with two factors (Mroczek et al., 1993; Myers and Steed, 1999), an option we adopted.

### Procedure

The sample consisted of gymnasts from the junior and senior categories present in the First Phase of the Spanish Cup of AGG of the 2018–2019 season and was selected with authorization from the Spanish Association of Aesthetic Group Gymnastics (SAAGG). The participating clubs had been contacted prior to the cup with information on the objectives of the investigation, anonymity, confidentiality of the data and informed consent, so that minors could bring a consent form signed by their parents or legal guardians to the championship. All gymnasts who decided to participate brought or signed (if they were of legal age) their consent forms and answered the questionnaire on the day of the competition between 30 and 60 min after the competition. Everyone received the same information through the protocol designed for this purpose: they were exposed to the objectives, reminded of confidentiality and given instructions on how to complete the questionnaire. The organization sponsoring the event arranged for a room attached to the competition zone to be used for the completion of the questionnaires. The questionnaires were administered by research personnel, who were trained for this purpose and present to explain the dynamics and answer any questions. The average time to complete the questionnaire was 10–15 min. The approval of the Research Ethics Committee of the University of Murcia was requested, which determined that the study, despite using human subjects, was observational and did not contain ethical aspects; therefore, it did not require the approval of the committee.

### Statistical Data Analysis

For the qualitative variables (marital status, educational level) the number of cases present in each category and their corresponding percentages were calculated. For the quantitative variables, the minimums, maximums, means, and standard deviations were computed. The Student's *t*-test was used for the comparison of the *means between two groups* after testing the assumption of normality with the Kolmogorov-Smirnov test and the assumption of variance homogeneity with the Levene test. The size of the effect was measured by *d* de Cohen, values below 0.2 indicate a small effect size, 0.5 of medium magnitude and 0.8 indicate a high effect size (Cohen, 1988). To study the possible relationship between two variables, Pearson's linear correlation coefficient (r) was *calculated*. The reliability of the scales was studied using the Cronbach *alpha* coefficient (α). A multiple linear regression model was developed to determine the possible effects of the demographic variables, competition category and years competing years and the scales of the motivating climate perceived in sport, total group cohesion and optimism on competitive anxiety. The statistical analyses were performed with the SPSS 25.0 program for Windows. The differences were considered statistically significant *p*< *0.05*.

## Results

Table 1 describes the dimensions of the different scales used in the study and their reliability values α. With regard to the motivating climate in both dimensions, the α is >0.85, whereas the α's for competitive anxiety range from 0.84 to 0.88, with an α of 0.88 for total team environment. Their respective dimensions range from 0.81 for social integration to 0.88 for integration with the task. The reliability of the scales is high, with values ranging from 0.81 and 0.88, with the highest consistency index being that of the Total Cohesion Equipment Environment scale (α = 0.889).

**Table 1:** Descriptive and reliability of scales.

	**Min.–Max**.	**Average (SD)**	**Alpha cronbach**
**Motivating climate**			
Involvement in the task	43–88	66.38 (6.82)	0.855
Involvement in the ego	19–62	34.75 (9.25)	0.869
**Competitive anxiety**			
Total	19–120	61.22 (17.64)	0.846
Somatic anxiety	5–40	20.39 (7.54)	0.882
Concern	8–40	27.85 (6.87)	0.868
Deconcentration	5–40	12.98 (6.65)	0.84
**Team environment**			
TOTAL cohesion	46–120	98.63 (12.37)	0.889
Social group attraction	3–30	24.97 (4.13)	0.861
Group attraction task	12–30	24.38 (3.46)	0.843
Social integration	7–30	24.61 (3.74)	0.816
Task integration	12–30	24.67 (3.32)	0.883
**LOT-R optimism**			
Total	3–23	14.6 (4.17)	0.853
Optimism	1–15	8.07 (2.57)	0.888
Pessimism	0–15	5.51 (2.89)	0.819

In terms of age and the different scales, the Pearson's linear correlation coefficient values showed a statistically significant negative relationship with ego involvement (*r* = −0.228, *p* = 0.036), meaning that the older the gymnast, the lower the involvement of the ego. On the competitive anxiety scale, age showed a negative and statistically significant relationship with concern (*r* = −0.221, *p* = 0.038); therefore, older gymnasts exhibit less concern. A negative and statistically significant relationship was shown with the pessimism of the LOT-R scale (*r* = −0.221, *p* = 0.044), indicating that older gymnasts have less pessimism. No statistically significant relationships were observed on all other scales and dimensions.

In relation to the scores on the different scales according to marital status (single/married), as well as the results of the *t*-tests performed to compare the scores of single and married gymnasts, the results of the competitive anxiety scale showed that there are statistically significant competitive differences in the total score (*p* = 0.041) and in the dimension of concern (*p* = 0.006), resulting in the scores of the single gymnasts being significantly higher than those of the married gymnasts. No statistically significant differences were observed between single and married gymnasts on the rest of the scales and dimensions.

In relation to the categories the gymnasts were in, Table 2 provides the descriptions of the scores for the scales by sports category, as well as the results of the *t*-tests performed to compare the scores of the junior (13–16 years) and senior (>16 years) categories. For the competitive anxiety scale, the results showed that there are statistically significant differences in the total score (*p* = 0.021) and in the dimensions of concern (*p* = 0.001) and deconcentration (*p* = 0.052), where the scores of junior athletes (13–16 years) were significantly higher than those of senior athletes (>16 years). Considering that the effect size for ego involvement, total anxiety and worry is high and for deconcentration it is medium. No statistically significant differences between juniors and senior were observed for all other scales.

**Table 2:** Descriptive and comparative scores scales by category.

	**Category**		**Average difference**	***t*****-Test**		***d***
	**Junior**	**Senior**		***t*(38)**	***p*-value**	
**Motivating climate**						
Involvement in the task	66.12 (7.14)	66.52 (6.69)	−0.4	−0.273	0.786	−0.06
Involvement to the ego	39.03 (9.94)	32.44 (8.03)	6.59	3.323	**0.002**	0.75
**Competitive anxiety**						
Total	66.94 (17.72)	58.13 (16.95)	8.81	2.373	**0.021**	0.51
Somatic anxiety	21.32 (7.5)	19.89 (7.58)	1.43	0.896	0.374	0.19
Concern	30.68 (5.63)	26.32 (7.03)	4.36	3.326	**0.001**	0.66
Deconcentration	14.94 (7.78)	11.92 (5.74)	3.02	1.99	**0.052**	0.46
**Team environment**						
TOTAL cohesion	99 (9.78)	98.43 (13.64)	0.57	0.238	0.812	0.05
Social group attraction	25.56 (2.23)	24.65 (4.84)	0.91	1.261	0.21	0.22
Group attraction task	24.21 (3.23)	24.48 (3.6)	−00.27	−0.378	0.707	−0.08
Social integration	24.47 (3.38)	24.68 (3.95)	−0.21	−0.278	0.782	−0.06
Task integration	24.76 (2.94)	24.62 (3.53)	0.14	0.216	0.829	0.04
**LOT-R optimism**						
Total	14.2 (3.76)	14.79 (4.37)	−0.59	−0.674	0.503	−0.14
Optimism	7.94 (2.81)	8.14 (2.46)	−0.2	−0.353	0.726	−0.08
Pessimism	5.79 (2.9)	5.35 (2.9)	0.44	0.721	0.473	0.15

*Data in bold indicate significant differences with p < 0.05*.

**Table 3:** Correlations between scales.

	**Anxiety**	**Task involvement**	**Ego involvement**	**Cohesion**	**Optimism**
Anxiety	1				
Task involvement	−0.063	1			
Ego involvement	0.348^***^	−0.084	1		
Cohesion	0.094	0.493^***^	−0.204^*^	1	
Optimism	−0.163	0.265^**^	−0.209^*^	0.263^**^	1
Pessimism	0.312^**^	−0.123	0.429^***^	−0.008	−0.135

* *p < 0.05;*.

** p < 0.01;

*** *p < 0.001*.

When examining the possible relationships between competition years and the different scales used in the research (PMCSQ-2, SAS-2, GEQ, LOT-R), it was found by applying Pearson's linear correlation coefficient; that for the competitive anxiety scale, competition years showed a negative and statistically significant relationship with deconcentration (*r* = −0.215, *p* = 0.032), meaning that the greater the number of years of competition, the less deconcentration. On the other hand, the years of competition also had a statistically significant and negative relationship with the pessimism on the LOT-R scale (*r* = −0.207, *p* = 0.038), i.e., the more years of competition, the less pessimism. No statistically significant relationships were observed for all other scales and dimensions.

Table 3 shows the Pearson's linear correlation values between the different scales of the study. Anxiety correlates positively and statistically significantly with ego involvement *(p* < 0.001) and pessimism (*p* < 0.01). With regard to the scale for the motivating climate perceived in sport, involvement in the task correlates positively and statistically significantly with cohesion (*p* < 0.001) and optimism (*p* < 0.01), so high scores on involvement in the task are associated with high scores on cohesion and optimism. On the contrary, the involvement of the ego correlates negatively and statistically significantly with cohesion (*p* = 0.032) and optimism (*p* = 0.034), so high scores on ego involvement are associated with low scores on cohesion and optimism. Finally, cohesion correlates positively and statistically significantly with optimism, so high levels of cohesion are associated with high levels of optimism (*p* < 0.05).

To determine the possible effects of the demographic variables, competition category and years competing, and the scales of the motivating climate perceived in sport, total cohesion of the group and optimism in competitive anxiety, a multivariate linear regression model was developed, as shown in Table 4. The model was statistically significant [*F*_(7, 89)_ = 3.51, *p* = 0.002] and explained 21.7% of the variability in anxiety. The ego implication dimension of the perceived motivating climate scale in sport had a significant effect (*p* = 0.039), meaning that high levels of ego involvement are associated with high levels of anxiety. The rest of the variables did not show statistically significant effects.

**Table 4:** Linear regression.

	**B (ET)**	**Beta**	***T***	***p*-value**
Category (senior vs. junior)	−4.30 (4.36)	−0.117	−0.987	0.326
Years competing	−0.16 (0.59)	−0.03	−0.262	0.794
Task involvement	−0.28 (0.29)	−0.109	−0.983	0.328
Ego involvement	0.46 (0.22)	0.242	2.09	**0.039**
Cohesion	0.31 (0.16)	0.22	1.945	0.055
Optimism	−0.82 (0.69)	−0.119	−1.18	0.241
Pessimism	0.99 (0.67)	0.162	1.472	0.145

*B, Non-standardized regression coefficient; ET, Typical error; Beta, standardized regression coefficient. Data in bold indicate significant differences with p < 0.05*.

## Discussion

The objective of the research was to assess the perceptions about the psychological variables of cohesion and motivational climate that AGG gymnasts have about the context created by the coaches in their teams and to analyze in the different categories the optimism and competitive anxiety they face in competitive situations.

The lower the sports category, the greater the anxiety (h1). This is corroborated by previous studies by Hanton et al. (2008); Núñez and García (2017) and Mellalieu et al. (2004) Supported also by Pozo's (2007) study which assumes that athletes with greater competitive experience perceive anxiety as a facilitating factor. Presenting the highest (youth) scores on anxiety, according to a previous study by Hagan et al. (2017), this may be caused by the environment in which the athletes are immersed (i.e., a competitive environment). This is in contrast to Grossbard et al. (2009), who indicate that anxiety levels should be higher in the older categories compared to the younger ones, as the maturity and performance level is more demanding.

The greater the optimism, the less the anxiety (h2), is corroborated, supported by previous studies, as it has been shown by García-Naveira and Díaz (2010), that optimism is positively related to the performance of sportsmen and women, also in the line of Suárez (2018), indicating that optimism levels are inversely related to anxiety levels. This could be the case because the exposure to high stress stimuli such as competition generates more resilience for those sportsmen who present more optimism (Reche et al., 2014). Knowing that optimism is presented as a protective factor against anxiety [Pavez et al., 2012; Ortín et al. (2013)]. Since positive expectations about the future (optimism) increase efforts to achieve the goals (García-Naveira et al., 2015). Taking into account that optimism and the positive mood of the athlete are related to the effect of coping styles, which are the positive predictors of competition results (Wu and Dou, 2001; Qu et al., 2009).

The junior category in all dimensions reaches higher values than the data obtained in comparison with the senior category, since the novice or junior athletes, i.e., with less experience, tend to have more uncertainty caused by the unknown, which generates higher levels of anxiety before the competition (Vaca et al., 2017). Endorsed in previous studies with female athletes. Patel et al. (2010) and in young competitive football players García-Mas et al. (2009). Junior athletes show higher cohesion values than senior ones, and the effect of group cohesion in teams depends on the type of task demanded by the sport performed (Canto and Hernández, 2005). For Leo et al. (2010) it seems obvious that cooperation favors cohesion, with cooperation in the GGA being a fundamental factor in obtaining results, although it is estimated that social cohesion can have a positive influence on team performance. Group cohesion is fostered by a collective evaluation of events, in which each player feels he has a useful role within the team, reconciled with a healthy attributional style in the experiences of failure (Pietro et al., 2016).

Involvement in the task correlated positively and statistically significantly with optimism. This could be because knowledge of the expectations of peers and coaches about gymnasts allows them to create more positive and controlled attitudes, decreasing the climate of ego involvement and increasing task orientation Fournier et al. (2005). Circumstance already contrasted in previous studies Cantón et al. (2013) with football players and in the line of Aranzana et al. (2016) in swimmers. Significant positive results have been obtained with regard to the involvement of gymnasts in their work and cohesion. In relation to that, perceptions of the motivational climate act as good predictors of cohesion, especially when a climate of task involvement is created, which provides greater attraction and integration in the group in both dimensions (Balaguer et al., 2003). Besides, in the studies by Duda and Balaguer (2007) and López-Walle et al. (2011), sportsmen show more adapted motivational patterns when they perceive a climate of involvement in the task, confirming the importance of the social context in the motivation of belonging to a sports team as Eccles et al. (2003). On the contrary, Avalos et al. (2015) show that despite the perception of a task-oriented motivational climate, sportsmen and women give more importance to the achievement of a result. This is in contrast to the results of Beal et al. (2003), who found no relationship between cohesion and task involvement.

On the contrary, high scores in ego involvement are associated with low scores in cohesion, a circumstance favored by the sociomotor space where the technical exercise in AGG takes place, limited in a small (13 × 13 m) and closed area, endorsed by previous studies Quested and Duda (2009); Iglesias et al. (2019). This aspect was highlighted in previous research, where basketball, volleyball, and handball are more socially cohesive than sports that take place in a large area such as rugby and football (Rusu (2020), as well as in Castro-Sánchez et al. (2019b) with judocas.

This research found that, at high levels of cohesion, gymnasts show high levels of optimism (*p* = 0.005), finding no studies to ratify or refute this data. This circumstance is reinforced by the fact that cohesion is an emerging state that results from (and influences) other behavioral processes in which the team participates (McEwan and Beauchamp, 2014). This cohesion-optimism relationship is supported by the belief that the perceptions that individuals have about the cohesion of their team influence their behavior (Eys and Kim, 2017). This is highlighted in Bruner's et al. (2014) research where their findings linked cohesion to different cognitive skills among others, as well as Kao (2019) who determines that team cohesion and performance constitute a circular relationship of social skills. Evaluations of previous thoughts and emotions induced by the stress element of a competition, can be interpreted as facilitating or weakening elements for the athlete. This interpretation depends on the athlete's belief in his/her ability to cope and will also influence the athlete's future behavior (Neil et al., 2011).

### Conclusions

Positive perceptions of psychological variables for group cohesion have been confirmed in terms of older gymnasts that have been competing for longer periods, as well as the orientation of activity to the task as a proactive working method.

Competitive anxiety is reduced as gymnasts increase in age, have been competing for more years, and change their marital status to maintain a more stable personal relationship.

Individual optimism is associated with higher levels of cohesion between the group and activities directed at the task.

The safety provided by training to gymnastic mastery stems from the positive correlation between task-oriented work as a better model for greater group cohesion and greater optimism, based on years of training and experience in sports competition.

With regard to the contributions of this study, it is worth noting the uniqueness of the sample. All the gymnasts were nationally and internationally competitive, having participated in national, European, and world championships. Therefore, these findings are of importance to technicians and coaches.

### Study Limitations

As for the limitations of the study, first of all, we can point out that the data were self-reported. This is a common practice in studies but can lead to biases in participants' responses, exacerbating the variability and artificially increasing the correlations between variables (Spector, 2006). Secondly, the competition featured Spanish gymnasts and had its own cultural characteristics; therefore, the results cannot be extrapolated to other samples. Thirdly, we have used a cross-cutting and correlated design that, although common in research, continues to pose drawbacks; for e.g., it is impossible to establish causal relationships.

### Future Lines of Research

Training and competitive situations should be evaluated, and intervention programs that promote and improve the relationships within groups should be developed. The behaviors of, and models applied by, coaches in relation to their teams should be analyzed to generate training programs that indicate the importance and influence of their behavior in generating a motivational climate of involvement in the task. Studies on basic aesthetic gymnastics equipment should be deepened in order to adapt the practice to the characteristics of these age groups as well as enable the improvement of the relationships and interrelationships that are created within the team context. It would be interesting to carry out intercultural or transnational studies to see if the results of our work are similar to those of other countries.

## Data Availability Statement

The original contributions presented in the study are included in the article/supplementary material, further inquiries can be directed to the corresponding author/s.

## Ethics Statement

Ethical review and approval was not required for the study on human participants in accordance with the local legislation and institutional requirements. The patients/participants provided their written informed consent to participate in this study.

## Author Contributions

AM-M and CAM: conceptualization, validation, formal analysis, investigation, and supervision. FC-G and AD-S: methodology, resources, data curation, visualization and funding acquisition. AM-M: writing-original draft preparation and project administration. AM-M, CAM, FC-G, and AD-S: writing-review and editing. All authors: read and agreed to the published version of the manuscript.

## Conflict of Interest

The authors declare that the research was conducted in the absence of any commercial or financial relationships that could be construed as a potential conflict of interest.
